# Supplementary material for: TFE3 fusions drive oxidative metabolism and ferroptosis resistance in translocation renal cell carcinoma
Source: EMBO Mol Med. 2025 Mar 27;17(5):1041–70. doi: 10.1038/s44321-025-00221-7 (PMC12081665; doi:10.1038/s44321-025-00221-7)
Supplement: Supplementary file 1 — Appendix [file 44321_2025_221_MOESM1_ESM.pdf]

Appendix Figures

Appendix Figures 1: Expression of TFE3 and its fusion proteins in cell lines…………p2

Appendix Figures 2: Genes de-regulated by siTFE3 silencing..…………………………p4

Appendix Figures 3: Replicate TFE3 Cut&Tag using independent antibodies in UOK109 cells……………………………………………………………………………………...p6

Appendix Figures 4: Representative examples of TFE3 fusion protein binding at genes involved in OxPhos……………………………………………………………………..p8

Appendix Figures 5 : Immune infiltration and EMT in tRCC…………………………...p10

Appendix Figures 6 : TFE3 fusion regulation of ferroptosis gene expression……….…p12

Appendix Figures 7 : TFE3 fusion binding at the GPX4 and GCLC loci………………p14

Appendix Figures 8: TFE3 regulation of glutathion metabolism………………………p16

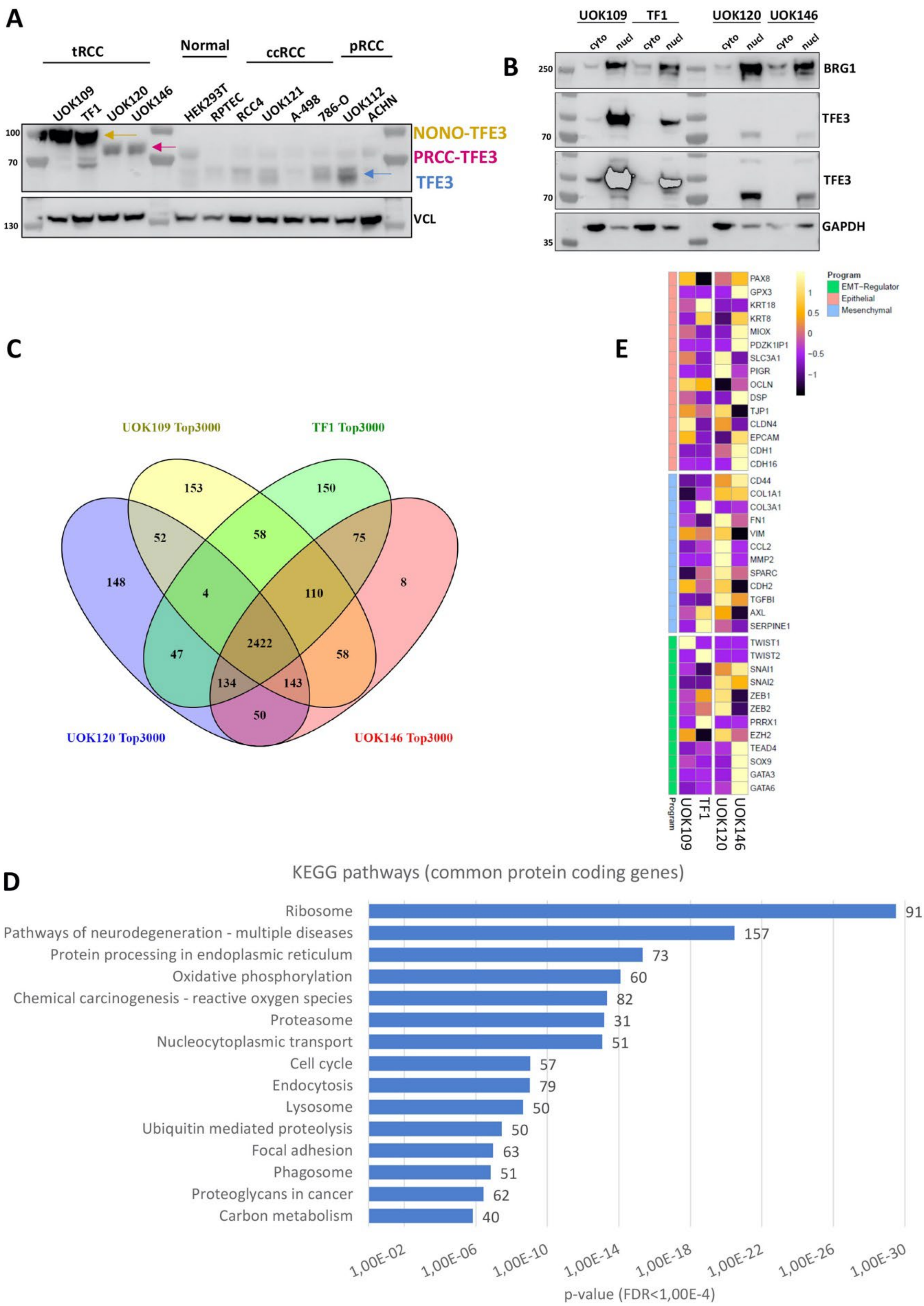

Appendix Figure S1

Appendix Figure S1. Expression of TFE3 and its fusion proteins in cell lines. A. Western blot analysis of total protein extracts from the indicated cell lines: tRCC (UOK109, TF1, UOK120, UOK146), 'normal' kidney (HEK293T, RPTEC), ccRCC (RCC4, UOK121, A-498, 786-O) and papillary RCC (UOK112, ACHN). Vinculin (VCL) was used as loading control. Blue arrow indicates native TFE3 protein, orange arrow NONO-TFE3 and pink arrow PRCC-TFE3 fusion protein. **B.** Western blot analysis of cytoplasmic (cyto) and nuclear (nucl) fractions from the tRCC cell lines. BRG1 was used as positive control for the nuclear extract and GAPDH for cytoplasmic extract. The same blot was exposed 1 second for NONO-TFE3 (upper panel) and 30 seconds for PRCC-TFE3 (middle panel). **C.** Venn diagram indicating overlap between the 3000 most expressed protein-coding genes in each tRCC line. **D.** DAVID gene ontology analysis of the common 2422 highest expressed protein-coding genes using KEGG. Each indicated KEGG pathway presented an FDR < 10^{-4} with associated p-value calculauted by hypergeometric distribution and Benjamini-Hochberg correction with the number of genes found in each pathway noted to the right of the bar. **E.** Heatmap showing the expression of selected epithelial or mesenchymal marker genes in each line.

A

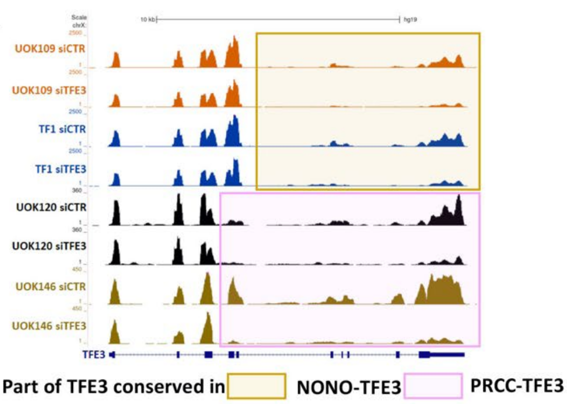

B

	Read count at fusion junction		Number of deregulated genes	
	siCTR	siTFE3	Up	Down
UOK109	72	22	186	299
TF1	119	35	598	400
UOK120	14	0	277	329
UOK146	151	11	288	356

C

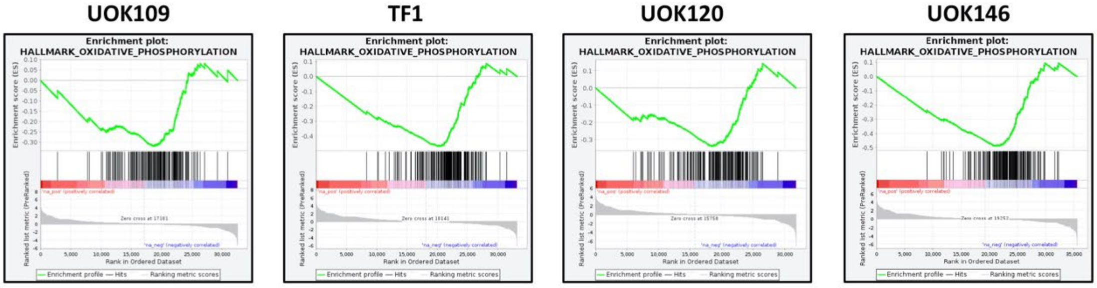

Appendix Figure S2

Appendix Figure S2. Genes de-regulated by siTFE3 silencing. A. UCSC view of the RNA-seq reads at the *TFE3* gene locus. Orange box represents the TFE3 region present in NONO-TFE3 fusion, while pink box encompasses the region conserved in PRCC-TFE3 fusion. **B.** Read counts at junction between TFE3 and the fusion partners and the number of deregulated genes in siTFE3 samples compared to siCTR (log2FC > ±1; adj.p.val<0.05). **C.** Enrichment plots of the Oxidative Phosphorylation (OxPhos) pathway after GSEA analysis between siCTR and siTFE3 conditions in each line.

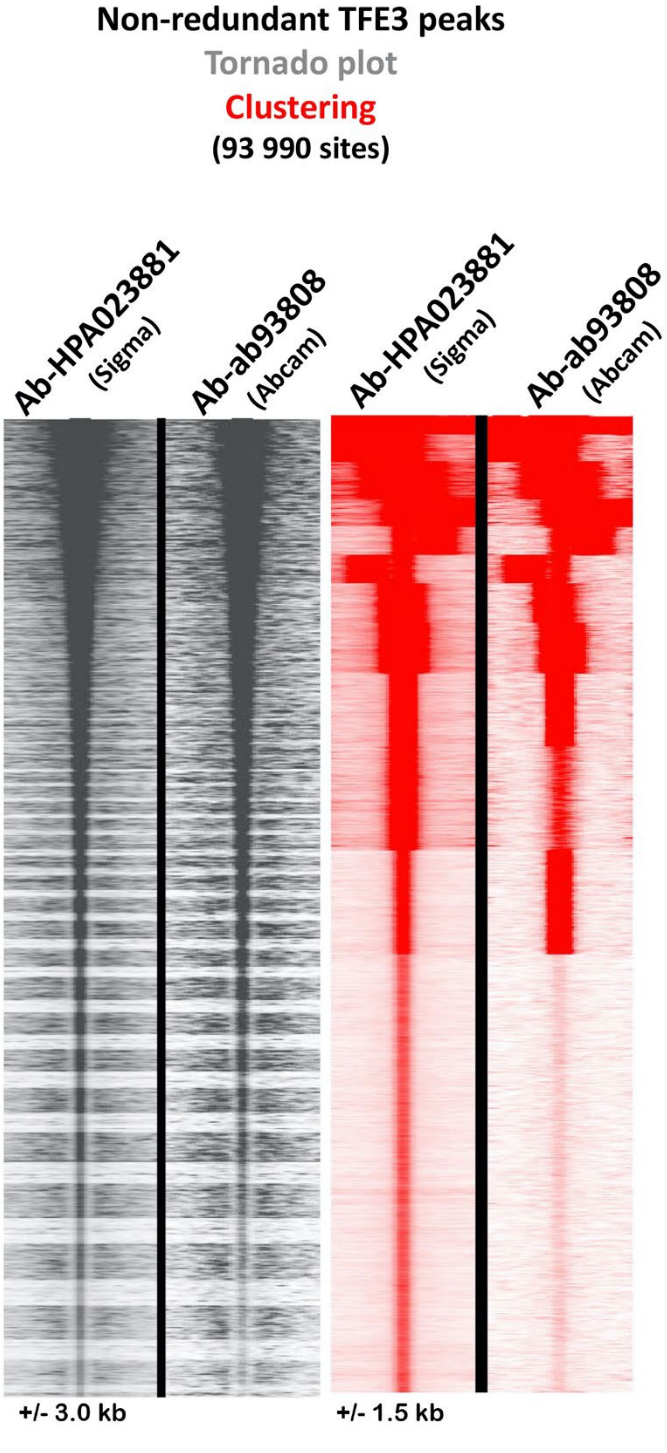

Appendix Figure S3

Appendix Figure S3. Replicate TFE3 Cut&Tag using independent antibodies in UOK109 cells. Tornado (left panel) and read density plots (right panel) of the replicate TFE3 Cut&Tag experiments with the antibodies shown above each track.

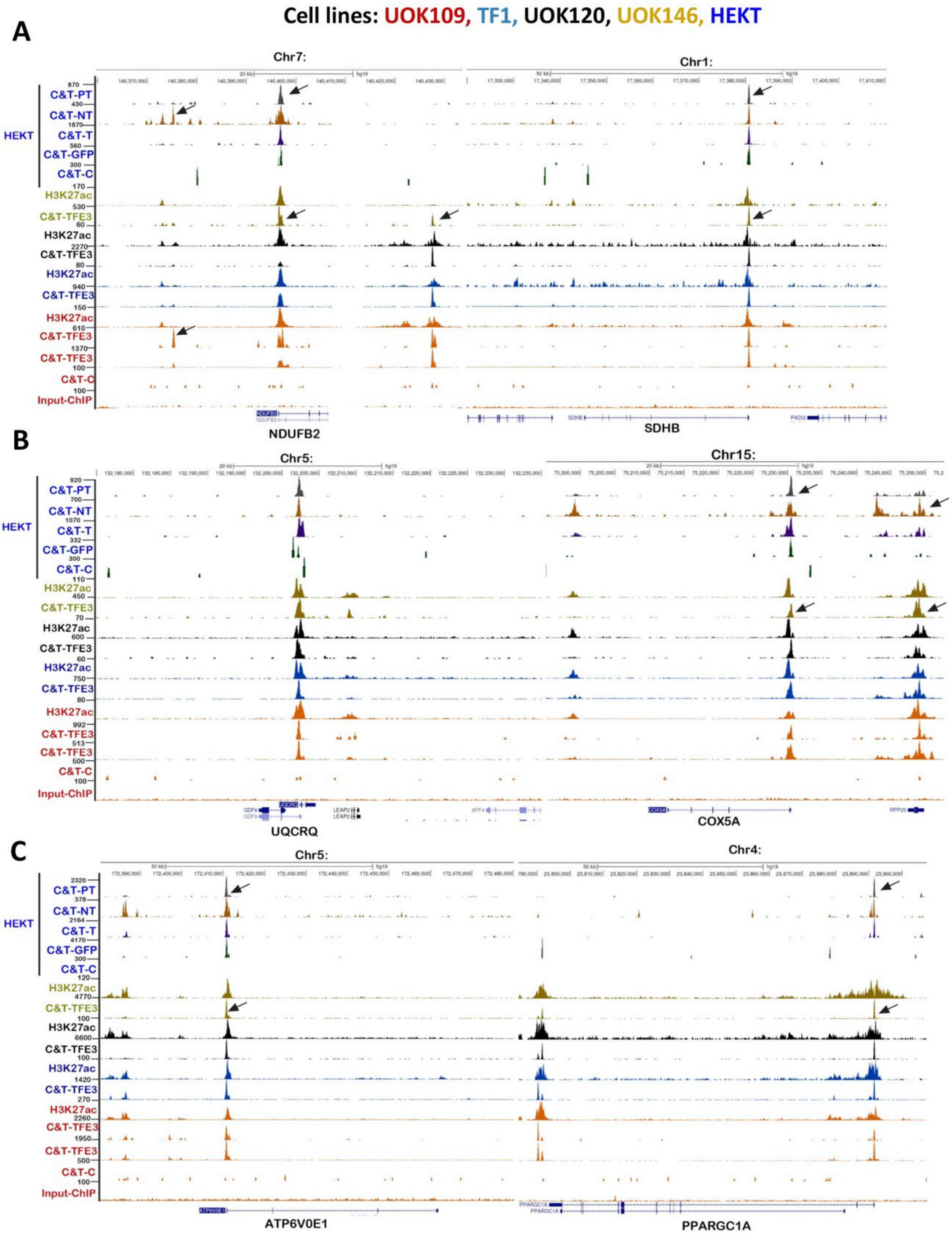

Appendix Figure S4

Appendix Figure S4. Representative examples of TFE3 fusion protein binding at genes involved in OxPhos. A-C. UCSC genome browser views of the TFE3 C&T, and H3K27ac ChIP-seq in each cell line, as indicated by the colour code, at the indicated loci: *NDUFB2* complex I (A, left panel); *SDHB* complex II (A, right panel); *UQCRQ* complex III (B, left panel), *COX5A* complex IV (B, right panel), the *ATPV6V0E1* (C, left panel) and *PPARGC1A* (C, right panel) gene loci. Arrows indicate TFE3 binding to H3K27ac-maked sites comprising an M-box motif.

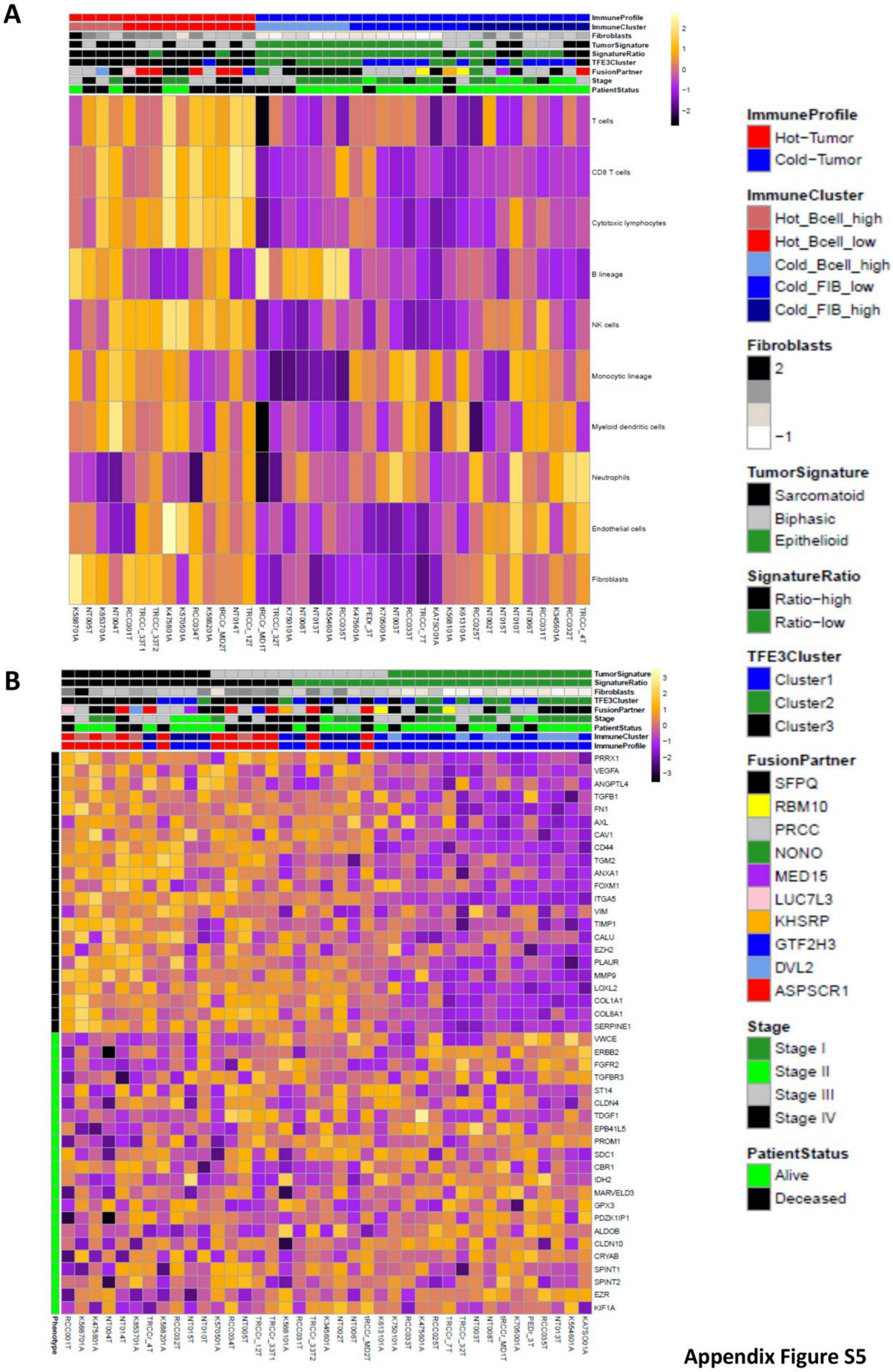

Appendix Figure S5

Appendix Figure S5. Immune infiltration and EMT in tRCC. A. MCP counter analyses of the tRCC cohort. **B.** Heatmap of the differential expression of a collection of epithelial and EMT related makers in the tRCC cohort displayed as a Z score.

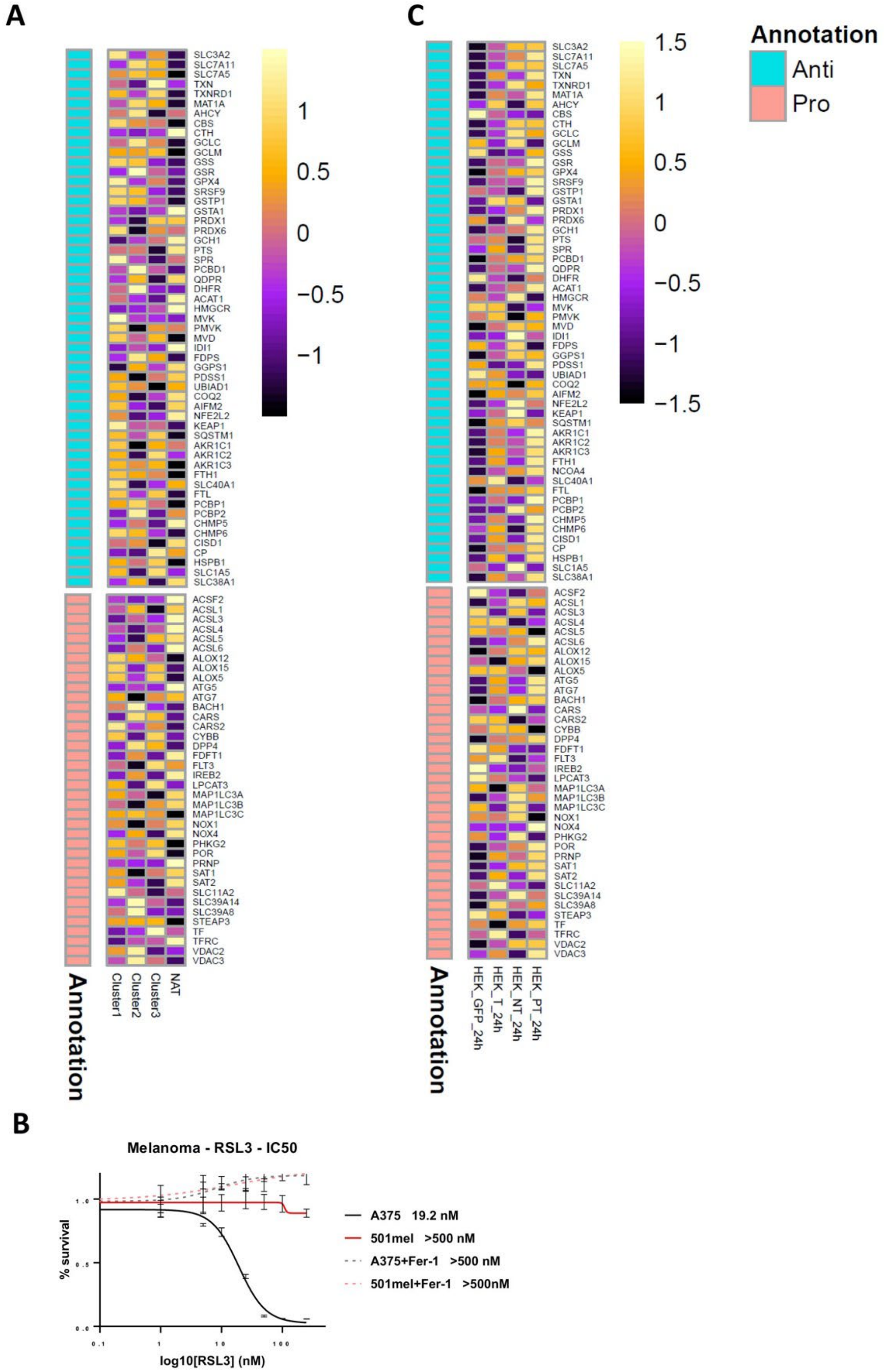

Appendix Figure S6

Appendix Figure S6. TFE3 fusion regulation of ferroptosis gene expression. A. Heatmap of expression of the indicated ferroptosis genes in the NAT compared to patient tRCC clusters 1-3. **B**. RSL3 IC50 values of control melanoma lines in presence or absence of 1 μM ferrostatin-1 (Fer-1) as indicated. **C**. Heatmap of expression of the indicated ferroptosis genes in the HEKT cells after 24 hours of Dox treatment.

Cell lines:

UOK109 ; TF1 ; UOK120 ; UOK146 ; HEKT

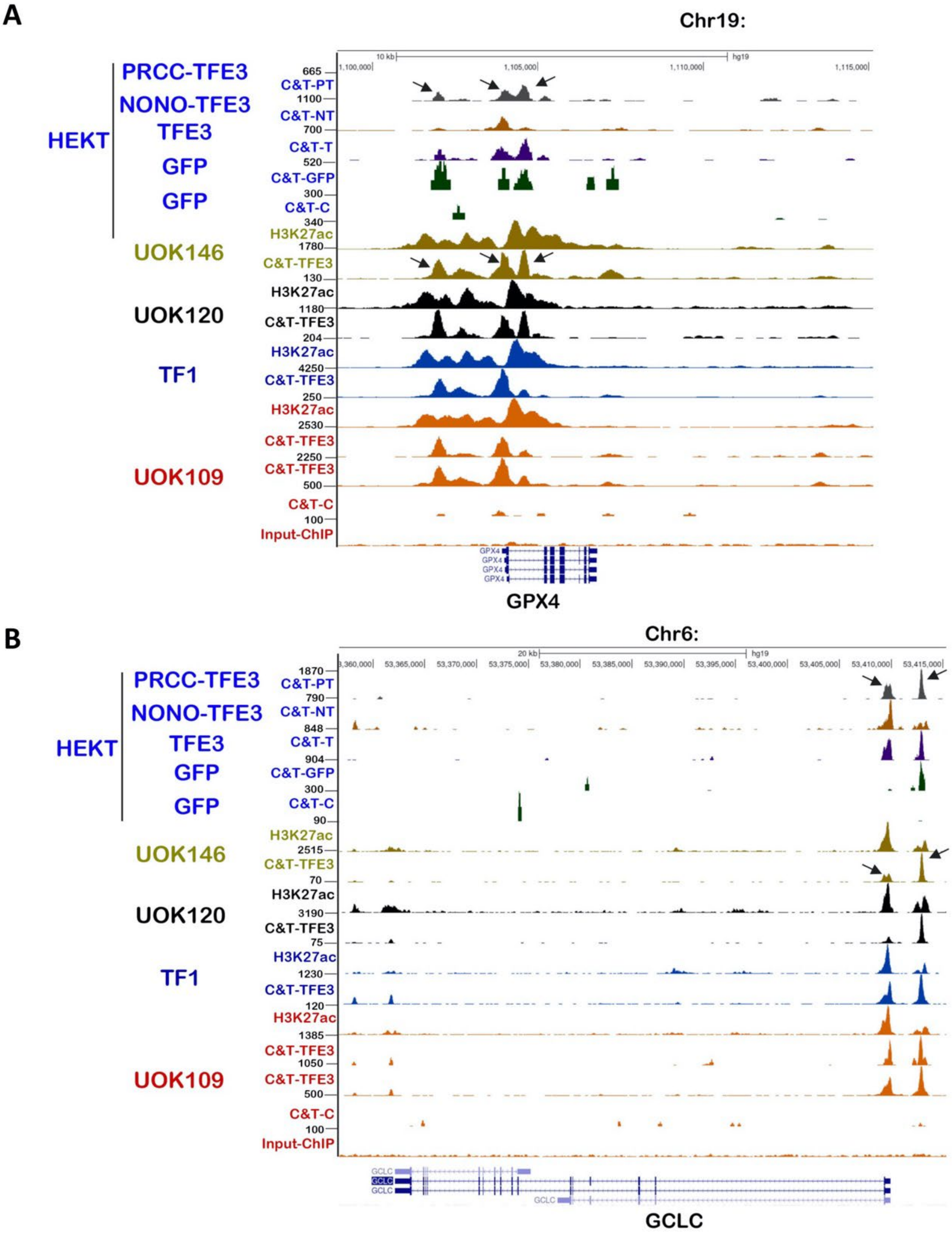

Appendix Figure S7

Appendix Figure S7. TFE3 fusion binding at the GPX4 and GCLC loci. A-B. UCSC genome browser views of the TFE3 C&T, ATAC-seq and H3K27ac ChIP-seq in each tRCC and HEKT cell line, as indicated by the colour code, at the GPX4 (A) and GCLC (B) loci.

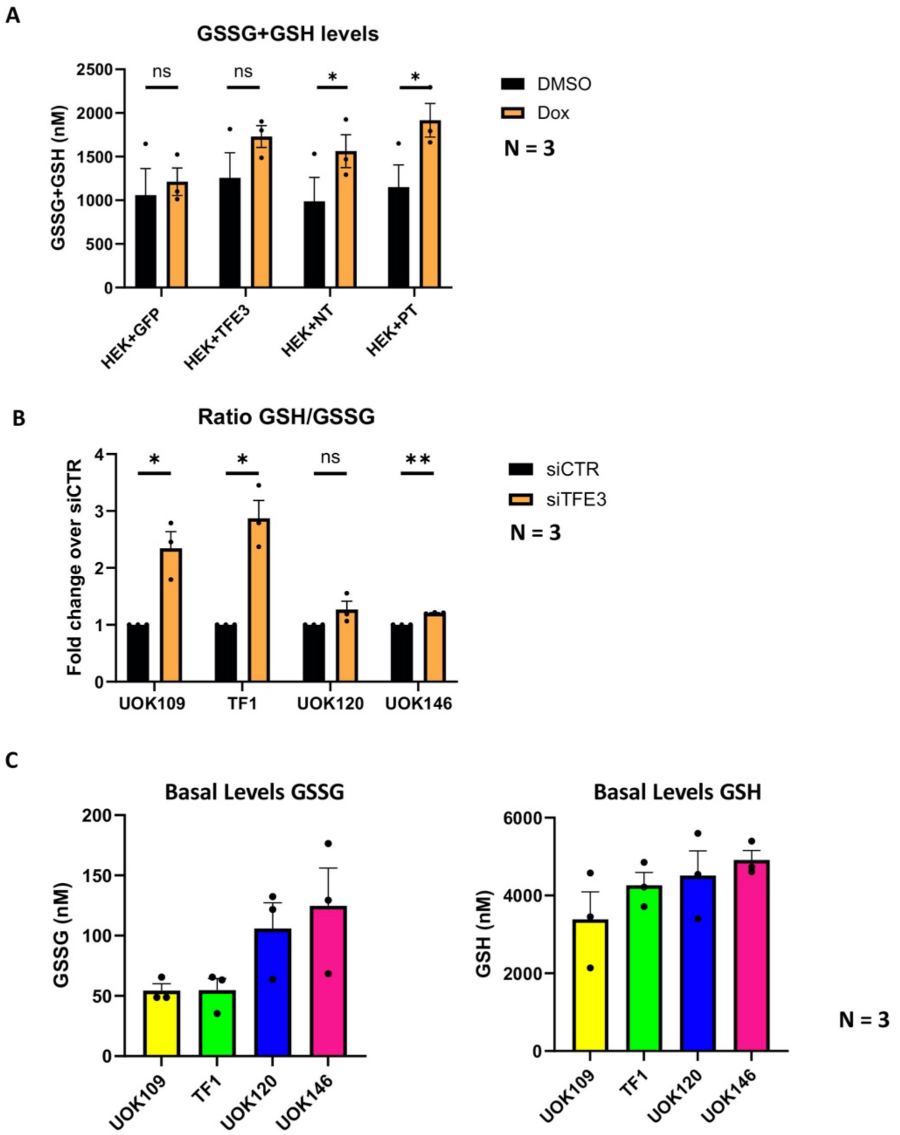

Appendix Figure S8

Appendix Figure S8. TFE3 regulation of glutathion metabolism. A. Total GSSG + GSH levels in the indicated HEKT cells. **B.** GSH /GSSG ratios in the indicated transfected tRCC cells. Note that given the much higher level of GSH compared to GSH in the HEKT cells, the changes in ratio were not significant despite the clear increase in the GSSG levels. **C.** Basal GSSG and GSH levels (nM) in siControl transfected tRCC cells. Paired T-test; * p.val<0.05, **: p.val<0.01, *** p.val<0.001, NS non-significant. Exact p-values UOK109 4.38E-02; TF1 2.7E-03; UOK120 2.10 E-02; UOK146 1.11E-03.
