# Supplementary figures and images for: TFE3 fusions drive oxidative metabolism and ferroptosis resistance in translocation renal cell carcinoma
Source: EMBO Mol Med. 2025 Mar 27;17(5):1041–70. doi: 10.1038/s44321-025-00221-7 (PMC12081665; doi:10.1038/s44321-025-00221-7)

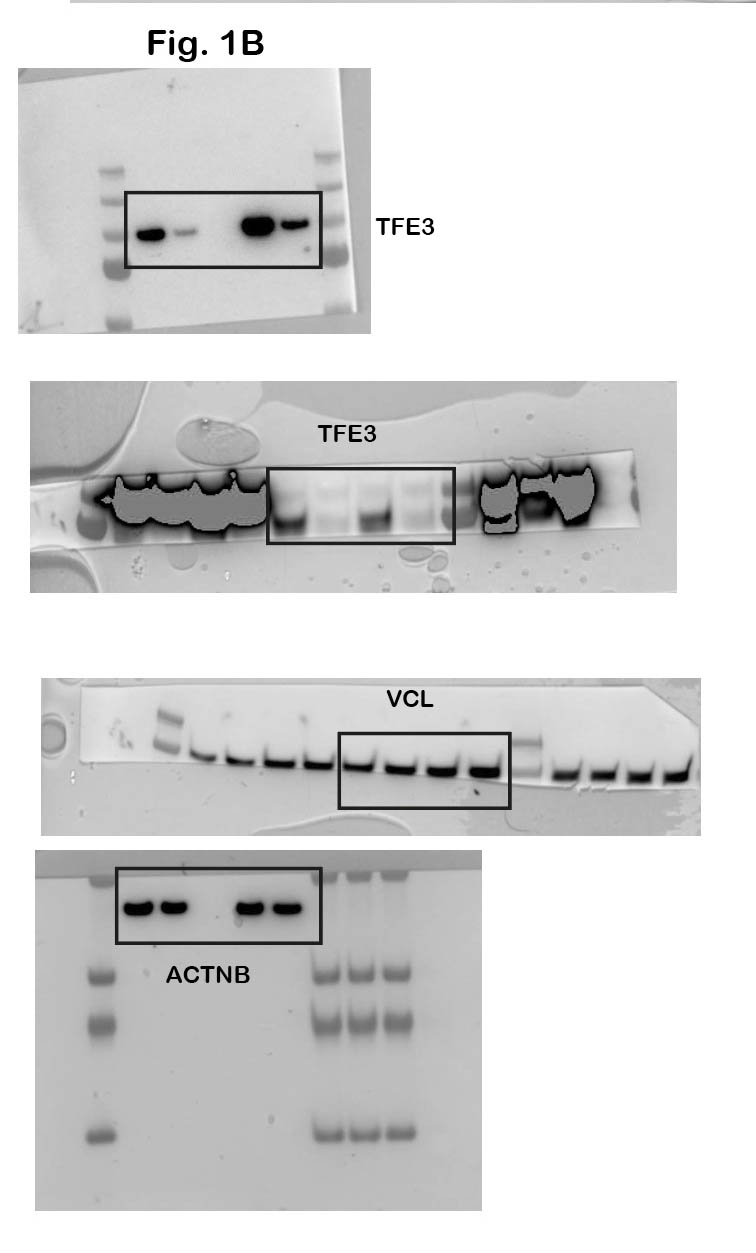

Supplement: Supplementary file 10 — Source data Fig. 1 [file 44321_2025_221_MOESM10_ESM.zip › Fig-1/Fig-1B.jpg]

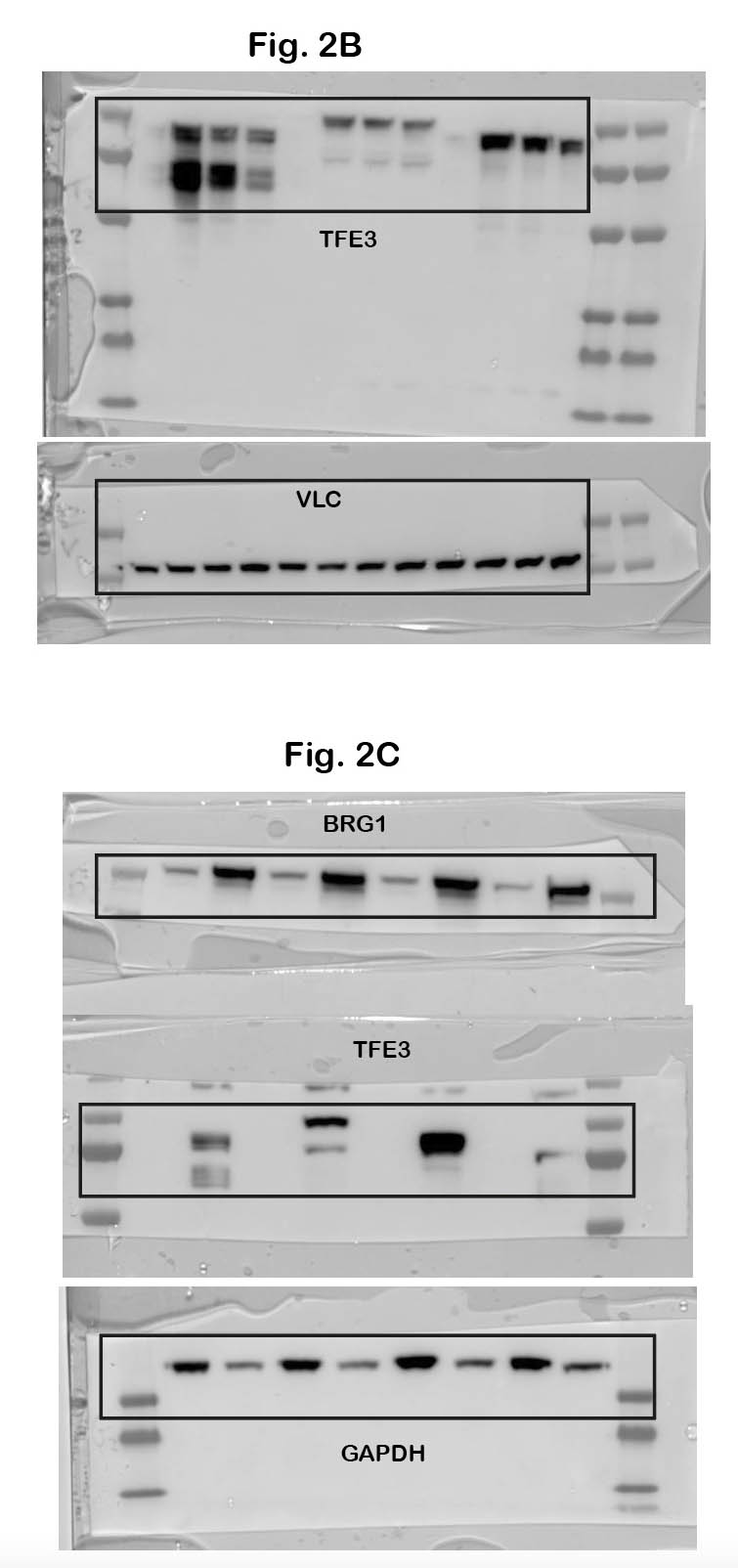

Supplement: Supplementary file 11 — Source data Fig. 2 [file 44321_2025_221_MOESM11_ESM.zip › Fig-2/Fig-2B-C.jpg]

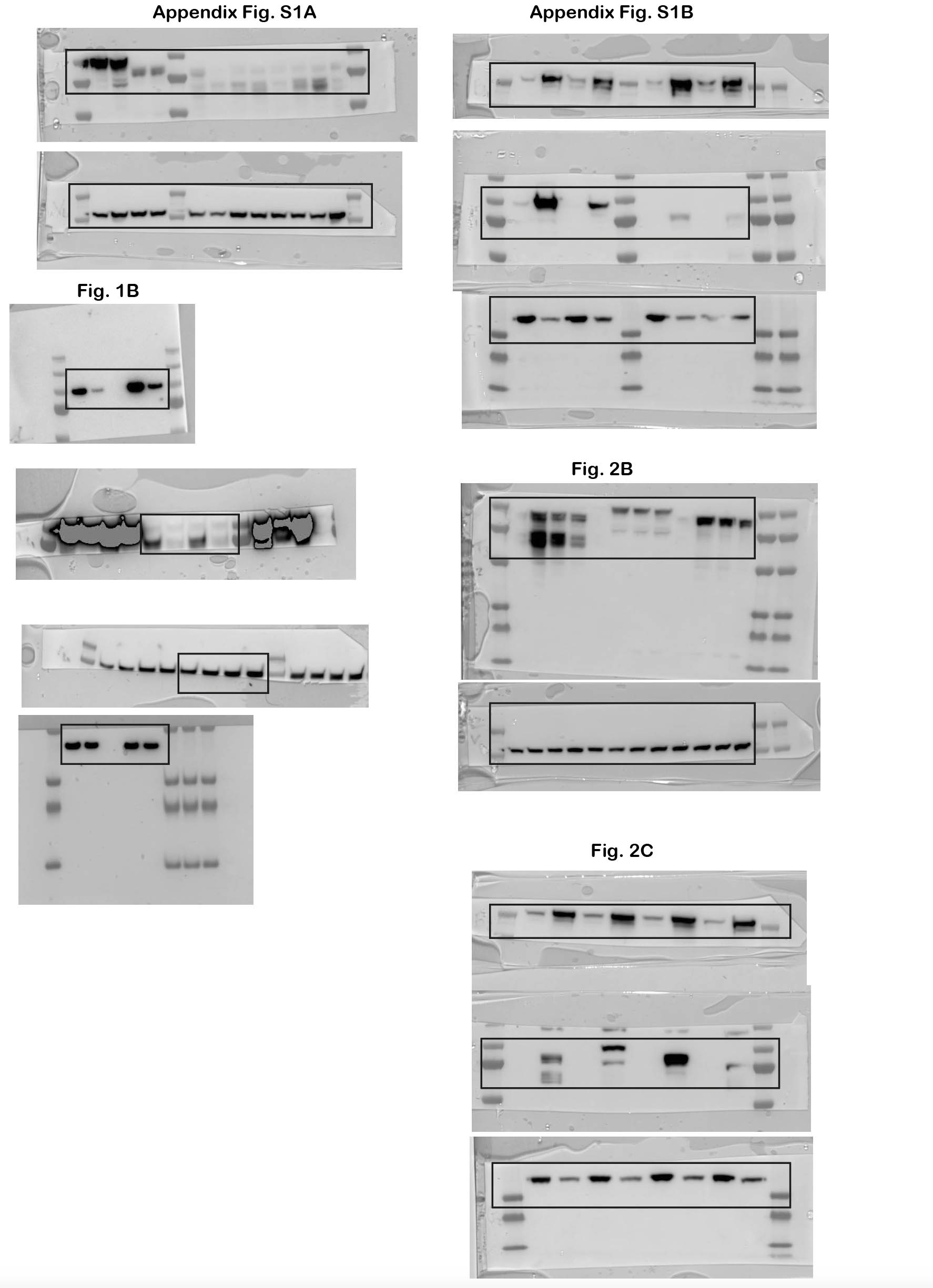

Supplement: Supplementary file 16 — EV and Appendix Figure Source data [file 44321_2025_221_MOESM16_ESM.zip › Fig EV and Appendix/Appendix Fig-S1/Appendix Fig-S1A-B.jpg]
