# Supplementary material for: TFE3 fusions drive oxidative metabolism and ferroptosis resistance in translocation renal cell carcinoma
Source: EMBO Mol Med. 2025 Mar 27;17(5):1041–70. doi: 10.1038/s44321-025-00221-7 (PMC12081665; doi:10.1038/s44321-025-00221-7)
Supplement: Supplementary file 17 — Expanded View Figures [file 44321_2025_221_MOESM17_ESM.pdf]

# Expanded View Figures

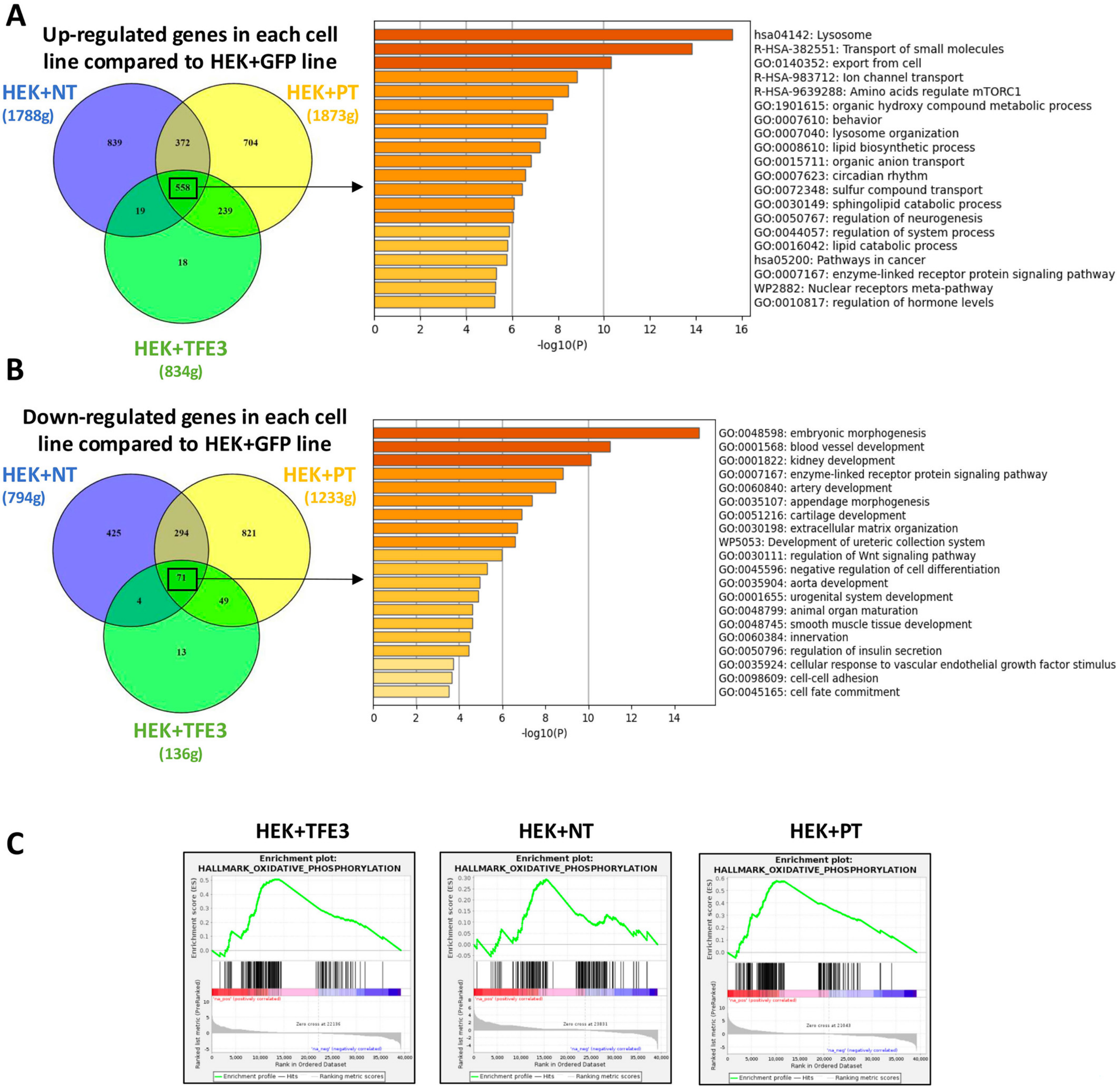


**Figure EV1. Genes regulated by ectopically expressed TFE3 fusion proteins.**

(**A**, **B**) Venn diagrams showing the number of deregulated genes in each line and the associated ontology analysis of the commonly regulated genes using MetaScape software with *p*-values calculated by an accumulative hypergeometric test. (**C**) Enrichment plots of the Oxidative Phosphorylation (OxPhos) pathway according to GSEA analysis with weighted Kolmogorov–Smirnov test after ectopic expression of native TFE3 or NT or PT fusion proteins in HEKT cells over GFP condition. Source data are available online for this figure.

© The Author(s)

A

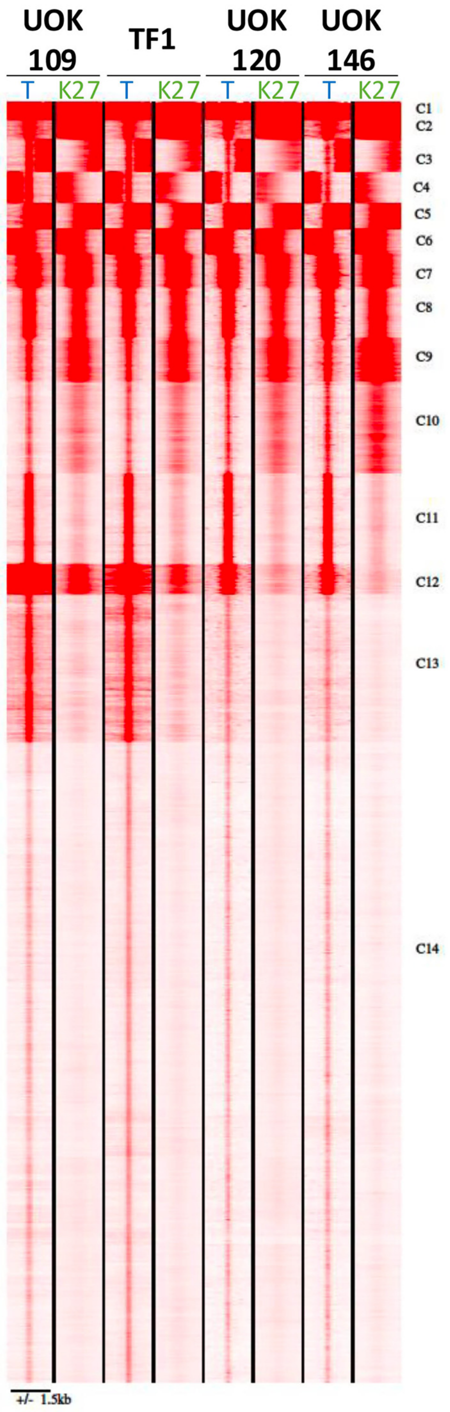


**Non-redundant TFE3 peaks**

**(169 896 sites)**

B

| Cell line | Cut&Tag TFE3 | ChIPseq H3K27ac | TFE3 with H3K27ac and E-box |
|---|---|---|---|
| UOK109 | 65901 | 44600 | 18466 (28%) |
| TF1 | 78598 | 56957 | 19919 (25%) |
| UOK120 | 33819 | 64545 | 11106 (33%) |
| UOK146 | 42760 | 51008 | 12714 (30%) |

C

| Cell line | Motif | | e-value | Distribution |
|---|---|---|---|---|
| UOK109 | SP2 (MA0516.3)<br>KLF5 (MA0599.1)<br>SP4 -MA0685.2) | 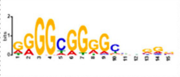 | 3.4e-126 | 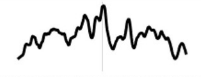 |
| | FOSL2 (MA0478.1)<br>FOSB::JUNB (MA1135.1)<br>FOS::JUN (MA0099.3) | 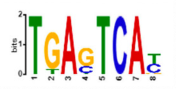 | 2.0e-120 | 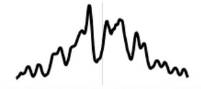 |
| | MITF (MA0620.3)<br>TFEB (MA0692.1)<br>TFEB_full | 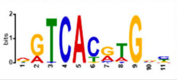 | 1.7e-53 | 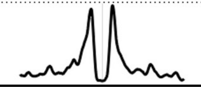 |
| TF1 | FOSL2 (MA0478.1)<br>FOSL2::JUNB<br>(MA1138.1) | 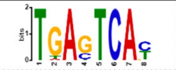 | 4.0e-164 | 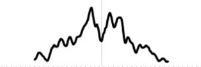 |
| | KLF12 (MA0742.2)<br>KLF10 (MA1511.2)<br>SP2 (MA0516.3) | 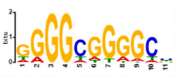 | 8.1e-84 | 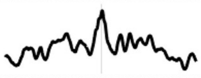 |
| | MITF (MA0620.3)<br>USF1_DBD<br>TFEB (MA0692.1) | 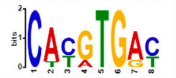 | 3.2e-46 | 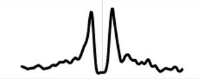 |
| UOK120 | TFEB (MA0692.1)<br>TFEB_full<br>TFE3_DBD | 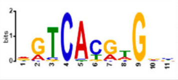 | 3.0e-618 | 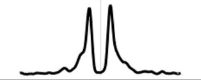 |
| UOK146 | TFEB (MA0692.1)<br>TFEB_full<br>TFE3_DBD | 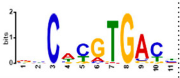 | 8.6e-587 | 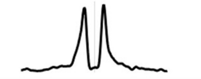 |

D

| Top1000 Peaks – RSAT analysis | UOK109 | | TF1 | | UOK120 | | UOK146 | |
|---|---|---|---|---|---|---|---|---|
| Motif | Rank | e-value | Rank | e-value | Rank | e-value | Rank | e-value |
| 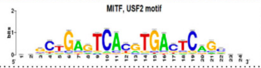 | 2 | 2.9e-61 | 6 | 3.4e-28 | 1 | 1.6e-66 | 2 | 6.9e-56 |
| 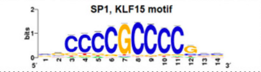 | 1 | 1.5e-79 | 1 | 5.6e-73 | 2 | 3.0e-54 | 1 | 3.6e-58 |
| 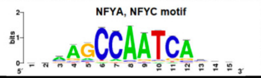 | 3 | 5.8e-29 | 3 | 7.6e-30 | / | / | / | / |



**Figure EV2. Profiling of TFE3 fusion protein genomic occupancy.**

(**A**) Read density maps for TFE3 fusion protein occupancy (T) and H3K27ac (K27) at all non-redundant sites. (**B**) Total numbers of peaks with the indicated characteristics in each cell line after removal of low-occupied sites (C14 of the density map in **A**). (**C**) Results of MEME-ChIP analyses of the DNA motifs at the top 1000 TFE3 bound sites in each cell line, with associated e-value and motif distribution over the peak. (**D**) Results of RAST analyses of transcription factor binding motifs at the top 1000 TFE3 bound sites in each cell line showing associated rank and e-value.

 © The Author(s)

**A**

| HEK293T Cell line | Cut&Tag TFE3 peaks | TFE3 peaks at TSS (500nt) |
|---|---|---|
| + GFP | 1058 | 615 (58%) |
| + TFE3 | 17250 | 9716 (56%) |
| + NONO-TFE3 | 28815 | 14690 (51%) |
| + PRCC-TFE3 | 20255 | 9707 (48%) |

**B**

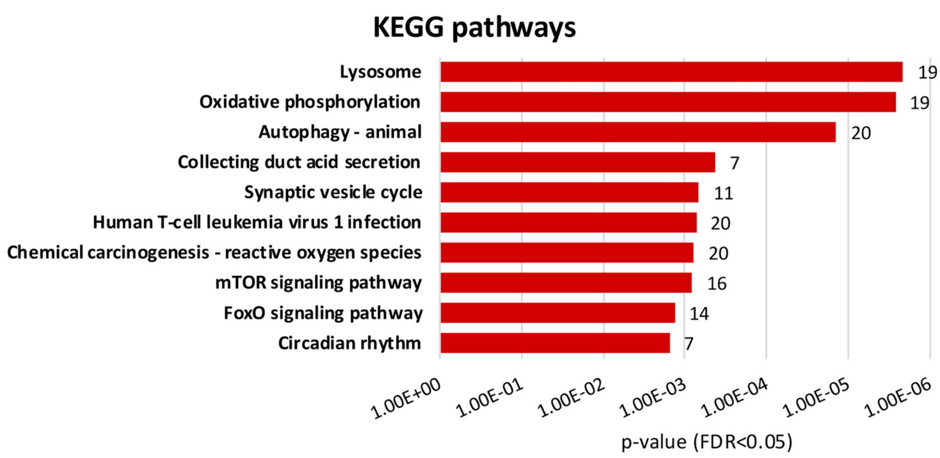


**C**

| Cell line HEK293T + | Motif | | e-value | Distribution |
|---|---|---|---|---|
| GFP | TFE3_DBD<br>TFEB (MA0692.1)<br>TFEB_full | 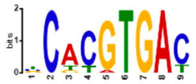 | 1.0e-184 | 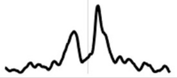 |
| TFE3 | KLF15 (MA1513.1)<br>KLF12 (MA0742.2)<br>KLF10 (MA1511.2) | 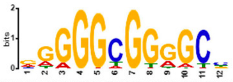 | 2.5e-203 | Not centrally enriched |
| TFE3 | TFEC (MA0871.2)<br>USF2 (MA0526.4)<br>Bhlhb2_primary (UP00050_1) | 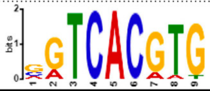 | 1.2e-54 | 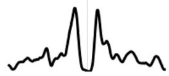 |
| NONO-TFE3 | KLF15 (MA1513.1)<br>SP2 (MA0516.3)<br>KLF12 (MA0742.2) | 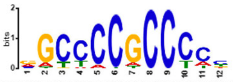 | 3.6e-428 | 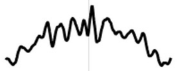 |
| NONO-TFE3 | NFYA (MA0060.3)<br>NFYC (MA1644.1)<br>NFYB (MA0502.2) | 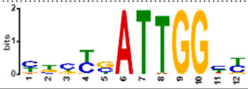 | 3.2e-330 | 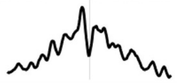 |
| PRCC-TFE3 | SP2 (MA0516.3)<br>KLF12 (MA0742.2)<br>KLF10 (MA1511.2) | 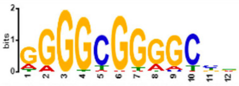 | 1.6e-196 | 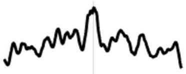 |
| PRCC-TFE3 | TFE3_DBD<br>TFEB (MA0692.1)<br>TFEB_full | 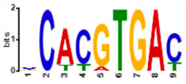 | 1.1e-91 | 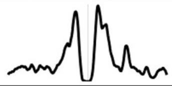 |

**D**

| Number of OxPhos genes common to the 4 tRCC lines With open-TFE3+H3K27ac-Ebox associated peak | |
|---|---|
| Among the 235 OxPhos-signature genes | 145 |
| Among the downregulated genes in siTFE3 (54) | 33 |
| Among the upregulated genes in HEK+ (59) | 44 |
| Among both downregulated genes in siTFE3 + upregulated genes in HEK+ (23) | 19 |

◀ **Figure EV3. TFE3 binding in HEKT cells.**

(**A**) Total numbers of peaks and those at the proximal promoters (500 nt from the TSS) in the indicated HEKT cell line. (**B**) KEGG ontology analyses of genes with binding of endogenous TFE3 in the proximal promoter in the HEK-GFP line. Each indicated KEGG pathway presented an FDR < 0.05 and an associated *p*-value calculated by hypergeometric distribution and Benjamini-Hochberg correction with the number of genes found in each pathway was noted to the right of the bar. (**C**) Results of MEME-ChIP analyses of the DNA motifs at the top 1000 TFE3 bound sites in each cell line, with associated e-value and motif distribution over the peak. (**D**) Number of OxPhos genes in each category associated with TFE3 occupied M/E box containing a H3K27ac-marked regulatory elements common to each cell line. Source data are available online for this figure.

 © The Author(s)

A

| Clinicopathologic characteristics | Total (n=38) | M stage, n (%) | |
|---|---|---|---|
| Age, median (range) | 33.5 (3-65) | M0 | 29 (76.31%) |
| Gender, n (%) | | M1 | 6 (15.79%) |
| Male | 13 (34.21%) | M2 | 1 (2.63%) |
| Female | 25 (65.79%) | Mx | 2 (5.26%) |
| T stage, n (%) | | Stage, n (%) | |
| ≤T2 | 23 (60.53%) | I-II | 20 (52.63%) |
| ≥T3 | 15 (39.47%) | III-IV | 18 (47.37%) |
| N stage, n (%) | | Alive status, n (%) | |
| N0 | 24 (63.16%) | Alive | 24 (63.16%) |
| N1 | 6 (15.79%) | Dead | 14 (36.84%) |
| N2 | 4 (10.53%) | OS, median (range) (months) | 47 (8.33-231.17) |
| Nx | 4 (10.52%) | | |

B

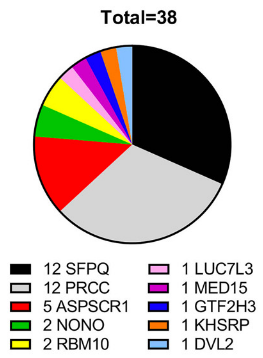


C

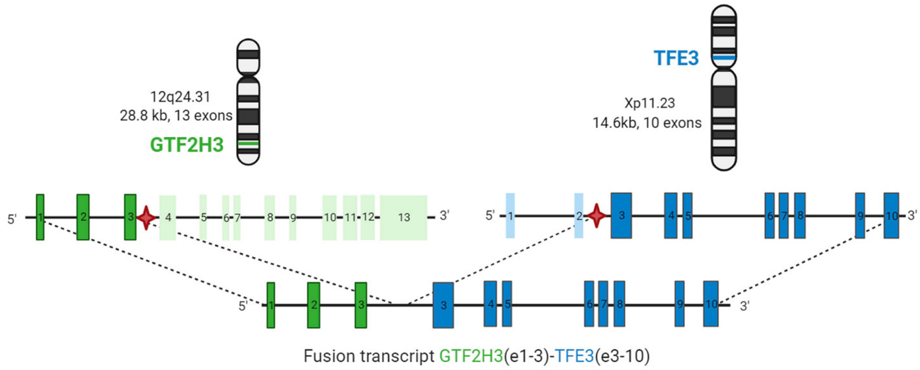


D

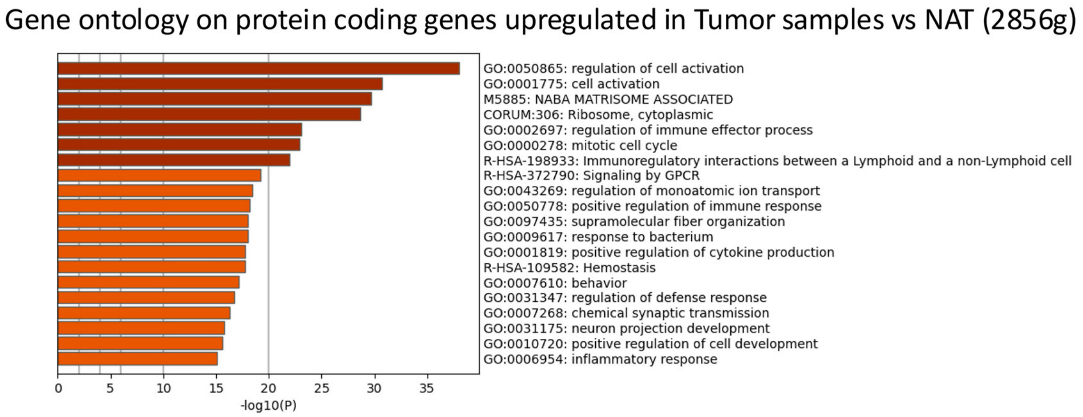


E

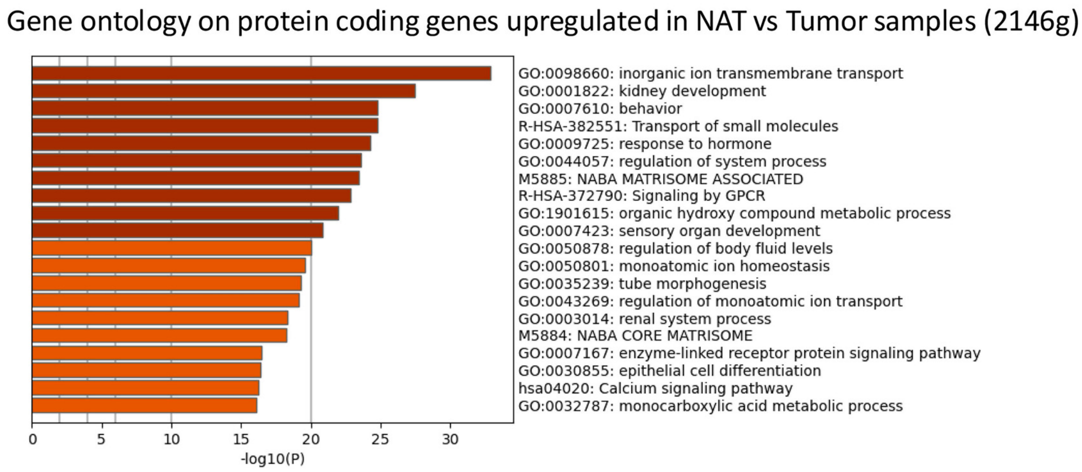


© The Author(s)

**Figure EV4. Clinical characteristics of the tRCC cohort.**

(**A**) Clinical and pathology characteristics of the tRCC patient tumor cohort. A full summary is presented in Dataset EV5. (**B**) Pie chart showing the contribution of the indicated fusion partners to the cohort. (**C**) Schematic representation of a novel *TFE3* fusion with *GTF2H3* encoding the p34 subunit of general transcription factor TFIIH. (**D**, **E**) Ontology of genes up- (**D**) or down- (**E**) regulated in tRCC tumors compared with NAT with *p*-values calculated by an accumulative hypergeometric test. Source data are available online for this figure.


© The Author(s)

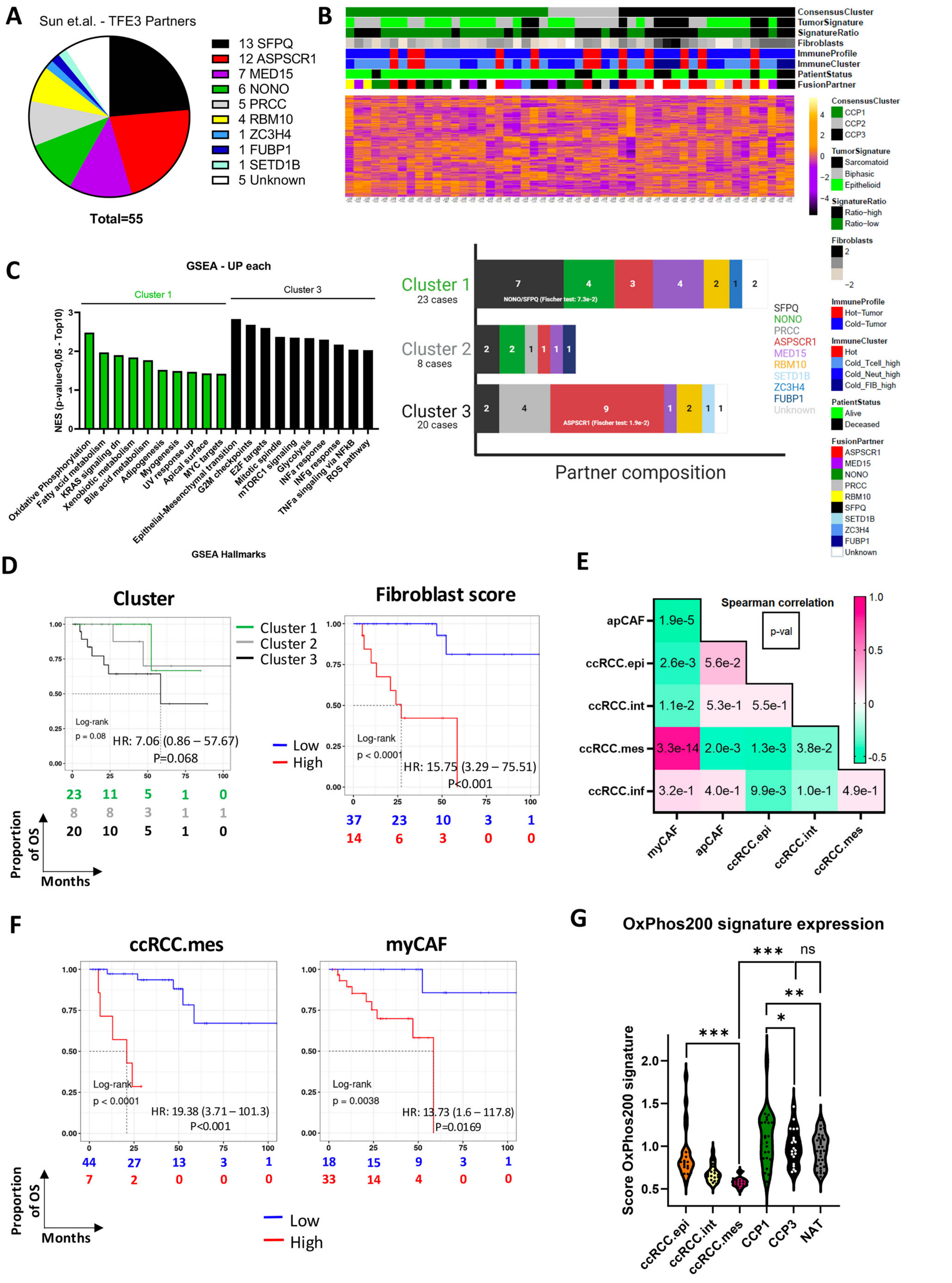
A
Sun et.al. - TFE3 Partners
13 SFPQ
12 ASPSCR1
7 MED15
6 NONO
5 PRCC
4 RBM10
1 ZC3H4
1 FUBP1
1 SETD1B
5 Unknown
Total=55
B
ConsensusCluster
TumorSignature
SignatureRatio
Fibroblasts
ImmuneProfile
ImmuneCluster
PatientStatus
FusionPartner
ConsensusCluster
CCP1
CCP2
CCP3
TumorSignature
Sarcomatoid
Biphasic
Epithelioid
SignatureRatio
Ratio−high
Ratio−low
Fibroblasts
ImmuneProfile
Hot−Tumor
Cold−Tumor
ImmuneCluster
Hot
Cold_Tcell_high
Cold_Neut_high
Cold_FIB_high
PatientStatus
Alive
Deceased
FusionPartner
ASPSCR1
MED15
NONO
PRCC
RBM10
SFPQ
SETD1B
ZC3H4
FUBP1
Unknown
C
GSEA - UP each
Cluster 1
Cluster 3
NES (p-value<0,05 - Top10)
Oxidative Phosphorylation
Fatty acid metabolism
KRAS signaling dn
Xenobiotic metabolism
Bile acid metabolism
Adipogenesis
Myogenesis
UV response up
Apical surface
MYC targets
Epithelial-Mesenchymal transition
G2M checkpoints
E2F targets
Mitotic spindle
mTORC1 signaling
Glycolysis
INFa response
INFg response
TNFa singaling via NFKB
ROS pathway
GSEA Hallmarks
Cluster 1
23 cases
NONO/SFPQ (Fischer test: 7.3e-2)
Cluster 2
8 cases
Cluster 3
20 cases
ASPSCR1 (Fischer test: 1.9e-2)
SFPQ
NONO
PRCC
ASPSCR1
MED15
RBM10
SETD1B
ZC3H4
FUBP1
Unknown
Partner composition
D
Cluster
Cluster 1
Cluster 2
Cluster 3
Log-rank
p = 0.08
HR: 7.06 (0.86 – 57.67)
P=0.068
23 11 5 1 0
8 8 3 1 1
20 10 5 1 0
Fibroblast score
Low
High
Log-rank
p < 0.0001
HR: 15.75 (3.29 – 75.51)
P<0.001
37 23 10 3 1
14 6 3 0 0
Proportion of OS
Months
E
Spearman correlation
p-val
apCAF 1.9e-5
ccRCC.epi 2.6e-3 5.6e-2
ccRCC.int 1.1e-2 5.3e-1 5.5e-1
ccRCC.mes 3.3e-14 2.0e-3 1.3e-3 3.8e-2
ccRCC.inf 3.2e-1 4.0e-1 9.9e-3 1.0e-1 4.9e-1
myCAF apCAF ccRCC.epi ccRCC.int ccRCC.mes
F
ccRCC.mes
Log-rank
p < 0.0001
HR: 19.38 (3.71 – 101.3)
P<0.001
44 27 13 3 1
7 2 0 0 0
myCAF
Log-rank
p = 0.0038
HR: 13.73 (1.6 – 117.8)
P=0.0169
18 15 9 3 1
33 14 4 0 0
Proportion of OS
Months
Low
High
G
OxPhos200 signature expression
Score OxPhos200 signature
*** ns
**
*
***
ccRCC.epi
ccRCC.int
ccRCC.mes
CCP1
CCP3
NAT

**Figure EV5. Clinical and molecular characteristics of an independent tRCC cohort.**

(**A**) Pie chart showing the contribution of the indicated fusion partners to the cohort. (**B**) Heatmap of the unsupervised clustering with the indicated clinical and molecular parameters showing the division of samples in 3 clusters (upper panel). Distribution of samples by fusion partner within the 3 major clusters (lower panel). (**C**) GSEA analyses of genes differentially expressed between clusters 1 and 3 indicating the Top 10 enriched terms (weighted Kolmogorov–Smirnov test *p*-value < 0.05, FDR < 0.05). (**D**) Kaplan–Meier curves for overall survival in patients according to the cluster or the fibroblast score, calculated from MCP-counter, using the optimal cut point method with the associated log-rank *p*-value and Hazard ratio (HR) from the univariate Cox proportional-hazard model. (**E**) Spearman correlation coefficient (colored box) and associated *p*-value (number in box) between the indicated populations after deconvolution using the ccRCC tumor cell and CAF signatures inferred by CIBERSORTx on bulk RNA-seq data from the tRCC tumor samples. (**F**) Kaplan–Meier curves for overall survival in patients according to ccRCC.mes or myCAF scores using the optimal cut point method with the associated log-rank *p*-value from Wald test and Hazard ratio (HR) from the univariate Cox proportional-hazard model. CcRCC.mes *p*-value 9.99E−07; MyCAF *p*-value 5.26E−06. (**G**) Expression score of the OxPhos genes in NAT, each tRCC cluster and the indicated ccRCC tumor types from the TCGA.KIRC collection. Overall scores were compared by Wilcoxon test (*: *p*.val < 0.05, **: *p*.val < 0.01, ***: *p*.val < 0.001) and are summarized in Dataset EV6. Exact *p*-values; ccRCC.epi vs ccRCC.mes 2.71E−09; ccRCC.mes vs CCP3 1.45E−11; CPP1 vs NAT 8.21E−03; CPP1 vs CPP3 3.28E−03; CPP3 vs NAT 7.28E−01. CcRCC.epi, ccRCC.int, ccRCC.mes $n = 20$; CCP1 $n = 23$; CPP3 $n = 20$; NAT $n = 18$. Source data are available online for this figure.

 © The Author(s)
